# Patterns of Care for Adolescent With Schizophrenia: A Delphi-Based Consensus Study

**DOI:** 10.3389/fpsyt.2022.844098

**Published:** 2022-03-30

**Authors:** Antonio Vita, Stefano Barlati, Antonello Bellomo, Paolo Fusar Poli, Gabriele Masi, Lino Nobili, Gianluca Serafini, Alessandro Zuddas, Stefano Vicari

**Affiliations:** ^1^Department of Clinical and Experimental Sciences, University of Brescia, Brescia, Italy; ^2^Department of Clinical and Experimental Medicine, University of Foggia, Foggia, Italy; ^3^Department of Nervous System and Behavior Sciences, University of Pavia, Pavia, Italy; ^4^Stella Maris, Scientific Institute of Child Neurology and Psychiatry, Pisa, Italy; ^5^Child Neuropsychiatry, Genoa and Department of Neuroscience (DINOGMI), IRCCS G. Gaslini Institute, University of Genoa, Genoa, Italy; ^6^Department of Neuroscience, University of Genoa, Genoa, Italy; ^7^Department of Biomedical Sciences, University of Cagliari, Cagliari, Italy; ^8^Department of Life Sciences and Publich Health, Catholic University and Bambino Gesù, Rome, Italy; ^9^Children's Hospital, Istituto di Ricovero e Cura a Carattere Scientifico (IRCCS), Rome, Italy

**Keywords:** adolescent schizophrenia, Delphi method, early diagnosis, expert consensus, pattern of care, treatment gaps

## Abstract

**Background:**

The current conceptualization of schizophrenia as neurodevelopmental disorder should lead to innovative public health policies in terms of a reorganization of the mental health care systems, particularly in the transition from adolescence to adulthood, to reduce personal, familiar, and social costs and burdens. The purpose of the project was to perform a survey among a panel of Italian schizophrenia experts, to share evidence-based information on adolescent schizophrenia and explore the degree of consensus among professionals in the following four macro-areas: early diagnosis; pharmacological treatment; health care system organization and transition process from adolescent to adulthood; and psychosocial interventions.

**Methods:**

The consensus process consisted of a two-step web-based Delphi method, which took place between June and November 2021. The survey was developed by a panel of four psychiatrists and four child neuropsychiatrists, identified as key opinion leaders (KOLs). The KOLs identified 21 statements involving a total of 70 items with a major need of clarification on early-onset schizophrenia (EOS). The survey was distributed to 86 specialists in psychiatry and child neuropsychiatry.

**Results:**

The results revealed a large agreement among the expert group on all the investigated areas of adolescent schizophrenia patterns of care and management. Consensus was ultimately reached for 67 items of the Delphi survey (95.7%), while negative consensus was reached for 2 items and no consensus was reached for 1 item.

**Conclusions:**

Overall, results showed a significant gap between the acquired scientific knowledge and clinical practice. In this scenario, it should be necessary to plan specific initiatives at a multiple level, to edit recommendations on clinical decision-making, as well as to prompt changes at the political and organizational levels, also involving scientific societies, patients, and family associations, to overcome the barriers that delay the implementation process.

## Introduction

Schizophrenia is a highly prevalent, severe mental illness, representing one of the main causes of years lost due to disability (YLD) in adults in Europe (1). Schizophrenia onset usually occurs in late adolescence or early adulthood, but it is frequently anticipated by a prodromal phase, in which cognitive impairment, negative symptoms and poor social functioning occur several years before the first episode of psychosis (2). Within the broader psychosis spectrum, childhood and adolescence schizophrenia are peculiar disorders (3). Particularly, in adolescent it is more often a severe and debilitating psychotic disorder with considerable impairments in psychosocial, educational, and occupational functioning with a heavy burden on health care services. Despite the relatively high (up to 5%) prevalence of psychotic symptoms in otherwise healthy children, childhood-onset schizophrenia (COS) is rare, so epidemiologic incidence data with diagnoses based on standardized clinical assessments are lacking. Since clinical, cognitive, genetic, and neuroimaging characteristics of early-onset schizophrenia (EOS) patients are, in part, outlined, current findings point toward continuity between the early and adult-onset schizophrenia, with the former possibly being a more severe variant of the latter (4), suggesting the needs to implement mental health systems to improve possible interventions, especially in the transition from youth to adult facilities. The purpose of the present paper is to provide an exhaustive evaluation of adolescent schizophrenia, covering epidemiologic, neurobiological, and clinical features, in order to identify feasible treatments plans, both pharmacological and psychosocial, reducing personal, familiar, and society burdens, and improving prognosis, psychosocial functioning, and quality of life (QoL). Moreover, the paper covers initial programs aimed to rethink mental health system in the transition from adolescence to adulthood. Specifically, a Delphi approach was used to share evidence-based information on adolescent schizophrenia and assess the degree of consensus among professionals in the following investigation areas: (i) early diagnosis; (ii) pharmacological treatment; (iii) health care system organization and transition process from adolescent to adulthood; and (iv) psychosocial interventions.

In the following section, we report a summary of the evidence provided by the scientific board, that lead to some brief and explicit statements on adolescent schizophrenia.

### A General Overview: Adolescence as a Major Risk Phase for Psychopathology

Schizophrenia is currently described as a neurodevelopmental disorder due to the interaction of different genetic and environmental factors that act long before the beginning of pathophysiological processes (5). Although psychotic onset is generally in early adulthood, less common in adolescence and quite rare in childhood, about 11–18% of patients present with their first episode of psychosis before the age of 18 (6, 7). Hence if schizophrenia peak onset is generally between 15 and 25 years of age (8), incidence rates increase around 14 years of age (9), with ~39% of male and 23% of female patients developing schizophrenia before 19 years of age (10). Schizophrenia before the age of 18 years is usually divided into two categories. Early onset schizophrenia (EOS) presents between the ages of 13 and 17 years, whereas very-early-onset schizophrenia (VEOS) presents at or before the age of 12 years. An early identification after the onset of psychotic symptoms is aimed at reducing possible adverse factors that determine poor psychosocial functioning and QoL in young patients (11–15). Among these factors, the duration of untreated psychosis (DUP), that is the time from the onset of psychotic symptoms to the commencement of adequate treatment, is one of the main issues (16). In this context, the early detection and treatment of help-seeking individuals and of those already in the earliest phases of a psychotic disorder is currently considered as the most promising strategy to improve treatment outcomes and long-term prognosis, and thus to reduce worst consequences of a full-blown psychotic disorder in youth populations (17).

### Adolescent Schizophrenia: Nosographic Background and Clinical Dimensions

If among children, schizophrenia is definitively a rare neuropsychiatric disorder with incidence rates at around 0.04% based on the observations from the National Institutes of Mental Health (NIMH) cohort (18, 19), adolescent schizophrenia is a devastating disorder, probably underdiagnosed and undertreated, characterized by greater clinical severity and worse outcomes compared to the adult-onset disorder (20, 21). In this scenario, although several definitions are currently applied to describe children and adolescent patients with early onset schizophrenia with no apparent consensus (22), some definitions should be highlighted. Based on age at onset, VEOS emerges before 12 years, while EOS starts before 17 years (23). VEOS was found to have a prevalence rate of 1/10,000, while EOS at around 1–2/100 (18). Since these operational definitions might be overlapping, two more strictly classifications are provided: COS is defined with an age of onset at 12 years or younger, while adolescent-onset schizophrenia is defined as beginning between 13 and 17 years (24). According to the scientific literature and for better understanding of the text, we will consider the terms EOS as adolescent schizophrenia and VEOS as COS (3). Despite these attempts in identifying the different forms of early-onset psychosis, at a nosological level patients with adolescent schizophrenia (EOS) or with COS (VEOS) are diagnosed using the same criteria as for adult-onset schizophrenia (AOS), following the Diagnostic and Statistical Manual [DSM-5; (25)] or the International Classification of Diseases, 10th Revision (26). This categorical approach could lead to not recognizing the juvenile variants of schizophrenia, with serious consequences for young subjects. From this point of view, a dimensional approach, rather than a categorical one, could favor early recognition and intervention of adolescent schizophrenia and COS (20, 27). At a developmental level, adolescent schizophrenia has been associated with poor premorbid functioning and developmental delays in reaching crucial milestones (28). At a clinical level, adolescent schizophrenia, compared to adult-onset schizophrenia is more commonly characterized by an insidious onset (21, 24), with prevalent negative symptoms (20, 29). Summing up these data, a systematic review, involving 1.506 patients with child or adolescent psychosis, showed peculiar clinical features in these samples: auditory hallucinations (81.9%), delusions (77.5%; mainly persecutory), thought disorders (65.5%), bizarre or disorganized behavior (52.8%), and negative symptoms (flat or blunted affect) (52.3%). Moreover, high comorbid rates such as posttraumatic stress disorder (PTSD) (34.3%), attention-deficit/hyperactivity disorder (ADHD), disruptive behavior disorders (33.5%), and substance abuse/dependence (32.0%) were also reported (30). In addition, cognitive impairment is a common feature of adolescent schizophrenia (31, 32), which occurs at the time of illness onset and once established, appears to be stable over time, without continued deterioration (33, 34). Regarding illness progression and outcomes, adolescent schizophrenia shows worse prognosis, with a severe and chronic course, so that only a minority of patients is able to get symptomatic remission and functional recovery (21, 35, 36). Taking these observations together, a recent systematic review confirmed that predictors of worse clinical, cognitive, and functional outcomes in adolescent schizophrenia are represented by premorbid difficulties, symptom severity at baseline (especially of negative symptoms) and longer DUP (37). Among these factors, current literature highlighted the association between greater clinical severity, worse outcomes, and longer DUP (38), that for adolescent schizophrenia is 3 to 5 times longer than the DUP in AOS (30).

### Adolescent Schizophrenia: Early Diagnosis

With these notions in mind, worldwide mental health policies are claimed to give priority to early intervention services for children and adolescents. Adolescent schizophrenia is grossly diagnosed with the same criteria of International Classification of Diseases (26) and Diagnostic and Statistical Manual [DSM-5; (25)], as used for adult patients. Thus, a good quality assessment should include a detailed history from all possible sources as well as a physical and mental state examination. In particular, psychotic symptoms dimensions, comorbid psychiatric and medical conditions such as developmental disorders (including speech and language difficulties), comorbid substance abuse, risk of harms/suicidality as well as psychosocial functioning, and socio-cultural milieu of the patient and family should be evaluated (17, 27, 39). Moreover, medical work-up is always needed with basic pediatric assessment (e.g., routine laboratory testing), neurological and cognitive examination, neuroimaging, and tests for specific medical syndromes (e.g., genetic, infectious, autoimmune, rheumatologic, metabolic, and toxicology screens) (39). However, clinicians should keep in mind that a definitive diagnosis of early-onset/adolescent schizophrenia requires time and repeated assessments since clinical presentations tend to change especially during the first few years of the psychotic disorder. Therefore, adolescent schizophrenia diagnosis should be made with caution and sensitivity as it is associated with significant negative psychosocial consequences, both for the patients and their caregivers (27, 39).

### Adolescent Schizophrenia: Treatment Options and Patterns of Care

As in AOS, treatment decisions in the adolescent schizophrenia population should be drawn by consulting all figures involved in the care of these patients, particularly, their caregivers, taking into account familiar perspectives and dynamics (17, 21). Clinicians should provide an integrated approach, including pharmacological, psychological, psychosocial, rehabilitative, and family-oriented interventions to address all the needs of the patients and their families (24, 27, 39). Admission at acute inpatient facility is recommended when the adolescent exhibits suicidal projects or attempts, self-injurious behaviors, aggression, severe agitation, poor general psychical conditions or when the familiar milieu is unsupportive or even hostile (39).

#### Pharmacological Interventions

At pharmacological level, antipsychotic agents are considered as the first line of treatment in the youth population. The main goals of pharmacotherapy interventions are to menage acute psychotic states and prevent relapse, always minimizing possible adverse events (24). Particularly, second-generation agents (SGAs) are typically offered as the first choice in early-onset/adolescent schizophrenia (27, 40, 41). Drugs like risperidone, aripiprazole, quetiapine, paliperidone, and olanzapine have actually received Food and Drug Administration (FDA) approval for treating schizophrenia in adolescents 13 years and older (42) since reductions of psychotic symptoms severity were observed compared to placebo (40). To date, in Europe only three SGAs - oral aripiprazole, oral paliperidone and lurasidone - have received approval from the European Medicine Agency (EMA) for treating adolescents with schizophrenia. Specifically, oral aripiprazole and oral paliperidone are recommended for adolescents aged 15 year and older. Since its positive efficacy and safety in adolescents - aged 13–17 years - with acute schizophrenia, lurasidone has been recently approved by EMA for adolescent schizophrenia starting from 13 years of age. Lurasidone favorable efficacy and safety profile in adolescent schizophrenia patients aged 13–17 years has also been demonstrated in the long-term period, through a 2-year extension study of the former (43). A recent network meta-analysis, including 28 randomized controlled trials (RCTs) with 3.003 participants, confirmed these findings, showing that olanzapine, risperidone, lurasidone, aripiprazole, quetiapine, paliperidone, and asenapine were significantly better than placebo, while haloperidol, trifluperazine, loxapine, and ziprasidone were not (44). Moreover, clozapine was found to be significantly more effective than all other included antipsychotics (44). However, clozapine should be proposed as a second-line agent in child and adolescent samples especially in treating refractory conditions: this is due to potential adverse events of clozapine, including risk of agranulocytosis, seizures, and metabolic disturbances (19, 45). Clinicians should keep in mind a higher risk of extrapyramidal symptoms (EPS), akathisia, prolactin elevation, sedation, cardiovascular effects (QTc prolongation, orthostatic hypotension, tachycardia, and pericarditis) and metabolic effects such as weight gain, dyslipidemia, glucose intolerance were reported in adolescent population rather than in adults, with polypharmacotherapy contributing to an increased risk (41, 46, 47). Although existing data indicate similar efficacy between first generation antipsychotics (FGAs) and SGAs (48), SGAs were also found to improve QoL and social functioning in adolescent schizophrenia samples (44). In particular, quetiapine and lurasidone were significantly more efficacious than placebo at improving QoL (43), while no significant effects were observed for asenapine and aripiprazole (44). On the other hand, risperidone, aripiprazole and lurasidone showed significantly better improvements in social functioning compared to placebo (44). However, despite the established efficacy, antipsychotic discontinuation is a common phenomenon also in adolescent schizophrenia patients. A national survey found that approximately 75% of the sample discontinued SGAs within 18 months of initiating treatment (49) and the Treatment of Early Onset Schizophrenia Spectrum Disorders Study (TEOSS) found that only 12% of subjects completed the 12-months period of taking their medications (50). Several reasons should explain this issue, mostly including presenting adverse events due to prescribed drugs (47). Thus, to limit this problem, clinician should keep in mind that FGAs are not recommended over SGAs in adolescent patients with schizophrenia due to increased risks of EPS and akathisia (27). On the other hand, a recent open-label study highlighted that, in the long-term, lurasidone in adolescents (13–17 years) with schizophrenia was associated with minimal effects on body weight, lipids, glycemic, and prolactin indices (43), while metanalytic evidence confirmed a reduced discontinuation rates for lurasidone over other antipsychotic agents (51). Guidelines recommend to carefully consider tolerability profile in the selection of an antipsychotic with a specific patient (51), taking into account several factors including tolerability drugs profile, patient and family preference, drug cost and availability (41, 47, 52).

#### Psychosocial Interventions

Since pharmacological treatments showed limited efficacy on negative and cognitive symptoms, and functional recovery in these populations (21), there is a growing interest in non-pharmacological approaches such as psychosocial interventions, although few data is available to date in adolescent schizophrenia patients. Adjunctive psychosocial interventions should be provided in combination with medications to reduce morbidity burden and promote treatment adherence and alliance (21). Preliminary evidence is provided for cognitive remediation (CR) efficacy in the first episode or in early schizophrenia, despite more research is needed to confirm the efficacy and the effectiveness of CR in the early course of schizophrenia (53). Concerning psychotherapy, although cognitive behavioral therapy (CBT) is an established treatment in AOS sample, only one report is currently available targeting early-onset patients (54). Only preliminary evidence was obtained with psychoeducation interventions (24), showing lower rates of rehospitalization in a small sample of adolescents with early onset psychosis (55). More recently, a RCT assessed the efficacy of a comprehensive psychoeducation problem solving intervention group in 55 adolescents with early onset schizophrenia and their parents (56). In this perspective, family-oriented interventions are particularly relevant during the early phases of the disorder (57), and current meta-analytic evidence confirmed the efficacy of family support interventions to reduce relapse and rehospitalization rates in this population (58). In this way, family support interventions resulted in improved caregivers' psychological health and general wellbeing and in reduced burdens of care (58).

### Health Care System Organization and Transition Process From Adolescent to Adulthood

In order to reduce personal, familiar, social costs and burdens, innovative public health policies are needed in terms of a practicable reorganization of the mental health care systems. A structural problem is that in most European countries, mental health care for children and adolescents with psychiatric problems is independent and operationally separated from that for adults (59). This type of organization, also reflecting differences in specific training programs for resident doctors, could lead to difficulties in the transition from adolescence to adulthood between mental health services, bringing the patients to receive less support in adult facilities and troubled changes in diagnosis and treatments (29). Thus, two alternative models of transition between child/adolescent and adult' services may be considered. The first model is based on the identification of a transitional team operating independently from youth and adult services: this model has been implemented in prevention and early intervention in psychosis programs, although the main weakness of this model is the introduction of additional splits within the system (29). Otherwise, the interlocking model requires the use of multidisciplinary care protocols interlocking child/adolescent and adults' services in which transition from these facilities is guaranteed by sharing all information and recommendations about several aspects of psychiatric pathways performed up to now (29). In Italy, the interlocking model was advised by the National Action Plan for Mental Health manifesto (60) providing recommendations in order to develop experimental projects aimed at prevention and early intervention. In particular, it was mostly recommended the creation of integrated and multidisciplinary teams, including both youth and adult mental health services, also involving families, educational facilities and environmental context. On the other hand, the ITAlian Partnership for Psychosis Prevention (ITAPP) project included five Clinical High Risk for Psychosis (CHR-P) academic centers across Italy, representing a promising template for transitional mental health services, aimed at early detection and intervention. In fact, serving both adolescents and young adults with multidisciplinary and integrated interventions, ITAPP project is aimed at developing specialized facilities that bridge the gap in the transitioning phase from youth to adulthood, thus ameliorating presenting symptoms, delaying, and preventing psychosis onset while reducing DUP (61).

### Objective

The purpose of this study was to perform a Delphi survey among a panel of Italian schizophrenia experts, in order to obtain a qualified consensus in managing patients with adolescent schizophrenia. In particular, the aims were to identify the characteristics of adolescent patients with schizophrenia, to define the best pathways for the management of schizophrenia in adolescent patients, especially in the transition phase, to understand the available psychopharmacological and psychosocial treatments and their impact on the patients' QoL, to identify patients' and caregivers' needs, and to find actions to address stigma.

The Delphi approach was used to share evidence-based information on adolescent schizophrenia and assess the degree of consensus among professionals in the following four macro-areas: (i) early diagnosis; (ii) pharmacological treatment; (iii) health care system organization and transition process from adolescent to adulthood; and (iv) psychosocial interventions.

## Materials and Methods

The Delphi method is a structured technique aimed at obtaining by repeated rounds of questionnaires a consensus opinion from a panel of experts in areas wherein evidence is scarce and opinion is important (62, 63). In the present manuscript, the consensus process consisted of a two-step web-based Delphi method, which took place between June and November 2021. The survey was developed by a panel of eight physicians (four psychiatrists, four child neuropsychiatrists) identified as key opinion leaders (KOLs) in their respective fields in Italy. The KOLs met to fully analyse the published literature and discuss the unmet needs about early-onset and adolescent schizophrenia. The first step of question sourcing served to collect an initial pool of feedback on areas that the KOLs considered most critical based on their clinical practice. Comments were then analyzed and coded into themes, grouping subsets of related items of enquiry. The organization of themes and items into a coherent and meaningful set of statements for the Delphi questionnaire was further informed by a literature review on the management of EOS. The first draft of the Delphi questionnaire was submitted to the KOLs for critical appraisal and improvement of the draft to ensure that items were relevant to the research question, were clearly worded and did not overlap with previous items. The KOLs identified 21 statements involving a total of 66 items with a major need of clarification, focused on the following topics: (i) early diagnosis; (ii) pharmacological treatment; (iii) health care system organization and transition process from adolescent to adulthood; and (iv) psychosocial interventions. Once developed, the survey was distributed to 86 specialists in psychiatry and child neuropsychiatry via an online survey platform with anonymized results. Panelists were psychiatrists and child neuropsychiatrists selected by the scientific board, working in academic and non-academic settings with solid experience in the field of schizophrenia (at least 5 years of clinical experience). The size of the expert panel was determined by involving specialists from 15 Italian regions, in order to have a representative sample of the national territory and a homogeneous distribution. Panelists were invited to rate their level of agreement or disagreement on each statement using a 5-point Likert scale, scored from 1 to 5 (1, extremely disagree; 2, disagree; 3, agree; 4, mostly agree; and 5, extremely agree). Results were expressed as a percentage of respondents who scored each item as 1 or 2 (disagreement) or as 3, 4, or 5 (agreement). A cutoff of 66% of agreement/disagreement was chosen a priori to represent positive or negative consensus, respectively. No consensus was reached when <66% of the answers fell in the same category (62, 63). In the first round of the Delphi survey, there were 70 respondents among the 86 invited panelists. For the statements and items on which consensus had not been achieved, panelists were asked to rate again in a second round their agreement/disagreement. The second round was completed by all the 70 panelists who responded to the first round. Table 1, in results section, shows demographic characteristics of responders. Descriptive statistics were performed to summarize the results.

**Table 1:** Characteristics of responders in the Delphi survey.

**Characteristics of responders (*N* = 70)**	Values*
Gender (female)	48.6%
Mean age (years)	51 ± 10.8
**Role**	
Hospital	67%
Territorial service	33%
Specialist with also academic role	21%
**Italian region**	
Northern Italy	51.4%
Central Italy	22.9%
Southern Italy	25.7%
**Years of experience**	
5–10	17%
11–20	33%
21–30	26%
>31	24%

*Values in table are presented as a percentage or as the mean ± standard deviation.

## Results

The first round of the Delphi survey had a response rate of 81.4%, whereas 100% of panelists who responded to round 1 completed also round 2. The average age of the respondents was 51 years, 34 (48.6%) were females, 34 (48.6%) were specialists in psychiatry and 36 (51.4%) in child neuropsychiatry. Table 1 summarizes the characteristics of responders in the Delphi survey, also including their role, years of clinical experience, and geographical distribution.

**Figure 1:**
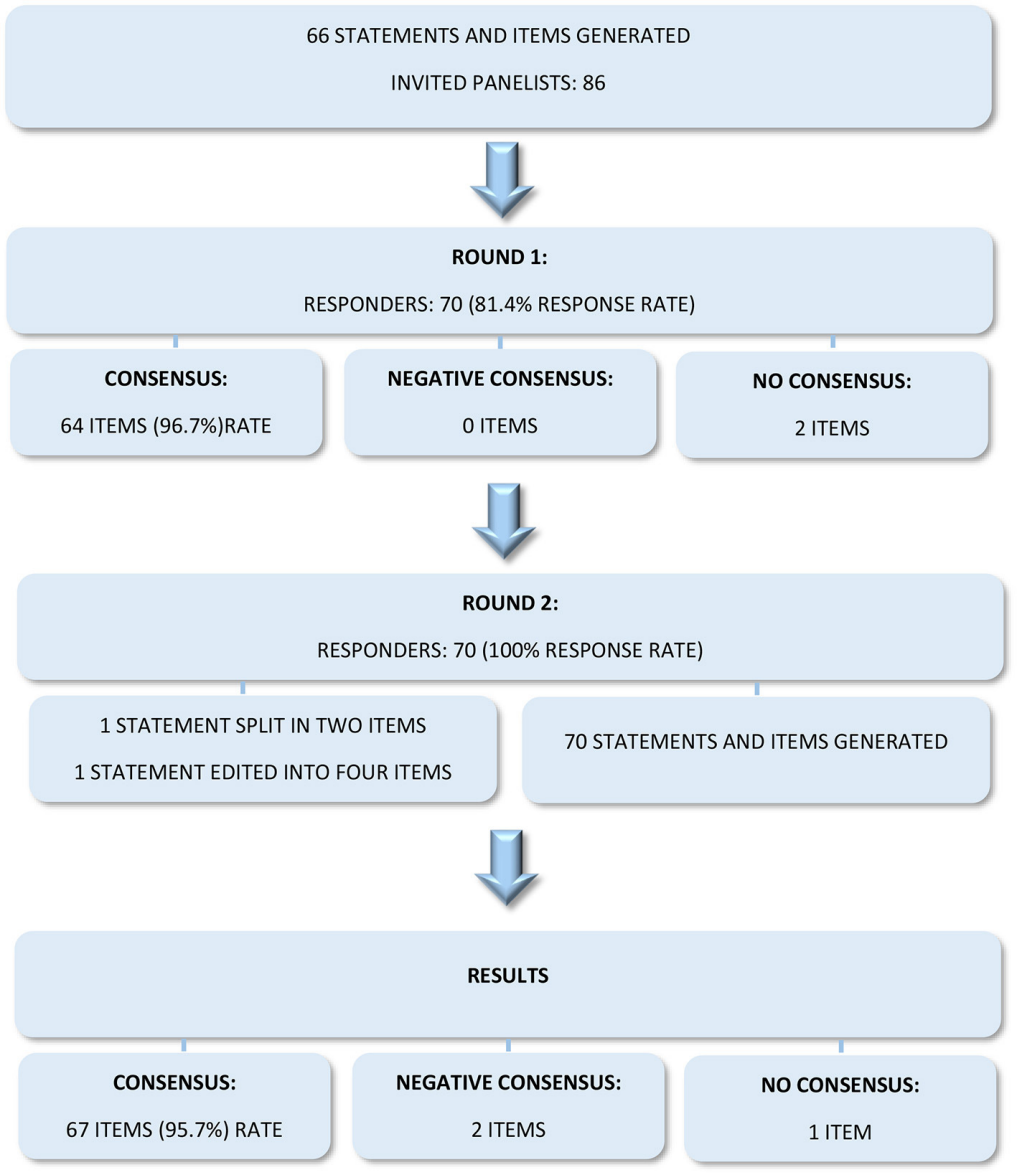
Delphi survey flowchart.

In round 1, consensus was reached for 64 of the 66 statements and items (96.7%), while no consensus was reached for two statements. The second round was performed on the two statements for which consensus had not been reached, after revising and clarifying the items. Specifically, one statement was split into two items, while the second one was edited into four clearer items, getting a final number of statements and items of 70. Overall, consensus was ultimately reached for 67 items of the Delphi survey (95.7%), while negative consensus was reached for 2 items and no consensus was reached for 1 item (Figure 1).

Tables 2.1, 2.2 summarize all the statements and items of the consensus, indicating the percentages of agreement and disagreement, mean, median, mode and standard deviation for each of them.

**Table 2.1:** List of consensus statements and Delphi survey results.

**Area of investigation**	**Statement/Item**	**Disagreement (score 1–2)**	**Agreement (Score 3–5)**
**EARLY DIAGNOSIS** – The importance of an early diagnosis of schizophrenia in youth: instruments, procedures, challenges (stigma), role of psychiatrists and patients/caregivers	1 – One of the most important objectives is the reduction of DUP, that is the period between the onset of psychotic symptoms and the moment in which the patient is adequately taken charge of with suitable treatment.	1%	99%
	2 – Significant evidence exists of a relationship between a prolonged DUP and the worst outcome of schizophrenia.	1%	99%
	3 – The prolonged and untreated state of active psychosis can have a neurotoxic action in the SNC.	1%	99%
	4 – The principal barriers of an early identification of a patient with SCZ in youth are:		
	4.1 - Lack/deficiency of dedicated human resources	7%	93%
	4.2 – Lack/deficiency of dedicated investments	4%	96%
	4.3 – Absence/lack of specific programs	16%	84%
	4.4 – Lack of trained operators	13%	87%
	4.5 – Stigma	16%	84%
	5 - Early intervention in subjects at high risk of schizophrenia or with prodromal symptoms is able to prevent or delay progression toward an overt psychotic disorder and to improve prognosis of the disorder as a whole.	3%	97%
	6 – In the diagnostic procedure course of SCZ in youth, it is necessary to investigate the presence of:		
	6.1 – Genetic vulnerability (first degree relatives affected)	1%	99%
	6.2 – Autism spectrum disorder	0%	100%
	6.3 – Communication (language) disorders	13%	87%
	6.4 **-** Intellectual disability	7%	93%
	6.5 – Attention deficit disorders/hyperactivity (ADHD)	14%	86%
	6.6 – Mood disorders	7%	93%
	6.7 – Social anxiety disorder	6%	94%
	6.8 – Symptoms of social withdrawal	0%	100%
	7 - A psychotic onset always requires hospitalization in a child/adolescent psychiatric hospital	41% (lack of consensus)	59% (lack of consensus)
	**Second round – revision:** 7a **-** A psychotic onset always requires hospitalization	70% (no consensus)	30% (no consensus)
	**Second round – revision:** 7b **-** Hospitalization for a psychotic onset in adolescence (or under 18 years of age) should always be carried out in a specific hospital dedicated to this age range	4%	96%
	8 – In order to reach an effective early diagnosis of schizophrenia in youth a diagnostic assessment with reliable and validated measures is needed	6%	94%
**PHARMACOLOGICAL TREATMENT** – Pharmacological treatment of schizophrenia in adolescents: criteria for the choice of antipsychotic therapy (symptom control, tolerability, psychosocial functioning, quality of life)	9 – The choice of treatment with antipsychotic drugs in adolescence should take into consideration:		
	9.1 - the clinical characteristics of the patient	0%	100%
	9.2 – the phase of the disease	4%	96%
	9.3 – eventual use simultaneously with substances of abuse	0%	100%
	9.4 – the presence of medical comorbidities (e.g., metabolic disorders)	1%	99%
	9.5 – the side effects of the drugs	0%	100%
	10 - The off-label pharmacological treatment of schizophrenia in adolescents should be based on the scientific evidence published in international journals.	4%	96%
	11 - Second generation antipsychotic drugs should be the first choice of treatment in patients with SCZ with onset in adolescence or in young adults.	0%	100%
	12 - The management of youth with SCZ should always include:		
	12.1 – Pharmacological treatment	4%	96%
	12.2 – Cognitive behavioral therapy	6%	94%
	12.3 – Psychosocial interventions for the patient	0%	100%
	12.4 – Psychoeducational interventions for family/caregivers	0%	100%
	12.5 – The possibility of treatment options having a low threshold or proximity treatment	6%	94%
	12.6 – Therapeutic options shared with families	0%	100%
	12.7 – Close collaboration between hospital and community psychiatrists	1%	99%
	13 – In the management of an antipsychotic treatment for adolescents with schizophrenia, it is always important to consider that:		
	13.1 – clozapine has demonstrated greater efficacy in treatment resistant	0%	100%
	13.2 – the choice of antipsychotic treatment should be based on its efficacy on positive symptoms	14%	86%
	13.3 - the choice of antipsychotic treatment should be based on the profile of the side effects	4%	96%
	13.4 - the choice of antipsychotic treatment should be based on its efficacy in improving cognitive symptoms and/or the psychosocial functioning	0%	100%
	13.5 – the perception of the subjective wellbeing of the adolescent with schizophrenia is, for the most part, influenced by tolerability of the antipsychotics rather than by their specific activity on psychotic symptoms	13%	87%
	14 - Patients with an initial psychotic episode have a good response even to low doses of antipsychotic drugs and tend to develop more side effects to pharmacological therapy	29%	71%
	15 – In the long term, pharmacological management of adolescent patients with SCZ should include:		
	15.1 – Individual periodic evaluation of the risks and benefits of the pharmacological therapy	1%	99%
	15.2 – Continuous use of the antipsychotic drug in order to prevent relapse, hospitalization and deterioration of the cognitive or psychosocial functioning	14%	86%
	15.3 – Use of the antipsychotics associated with a long-term good tolerability decreases the risk of side effects (sedation, cardiac toxicity, metabolic effects, endocrines, tardive dyskinesia, etc.), with negative effect on the compliance	0%	100%
	15.4 – Decrease the antipsychotic dosage only if conditions of sufficient clinical stability are guaranteed	3%	97%
	15.5 – Discontinuation of the antipsychotic therapy only if conditions of sufficient clinical stability are guaranteed	41% (lack of consensus)	59% (lack of consensus)
	**Second round – revision (15.5):** 15 – Over the long term, pharmacological management of adolescent patients with SCZ should include:		
	15.5.1 - Discontinuation of the antipsychotic treatment if the conditions of clinical remission are guaranteed for at least one year	76% (no consensus)	24% (no consensus)
	15.5.2 - Discontinuation of the antipsychotic treatment if the conditions of functional remission are guaranteed for at least 1 year	64% (lack of consensus)	36% (lack of consensus)
	15.5.3 - Discontinuation of the antipsychotic treatment if the conditions of clinical remission are guaranteed for at least 2 years	29%	71%
	15.5.4 - Discontinuation of the antipsychotic treatment if the conditions of functional remission are guaranteed for at least 2 years	21%	79%
	15.6 – Continuous use of psychosocial treatment independently of the use of that drug	0%	100%
	15.7 – In the case of discontinuation of the pharmacological treatment therapy, continuous clinical monitoring should, however, be guaranteed	1%	99%
	15.8 – Discontinuation of pharmacological treatment after a period of between 1 and 2 years after symptomatic remission presents a greater risk of relapse and hospitalization, and this risk should be discussed with the patient and the family.	7%	93%
	16 – Switching to another antipsychotic in the treatment of adolescent patients affected by schizophrenia:		
	16.1 – it should be considered only in the case of scarce tolerability and/or reduced efficacy	10%	90%
	16.2 – The use of antipsychotics with a more favorable profile should always be taken into account	1%	99%
	16.3 – it should always be taken into account in patients with medical comorbidities	3%	97%
	16.4 – it should always be carefully evaluated in the case of increased self-injurious/suicidal risk	1%	99%
**ORGANIZATION OF SERVICES/TRANSITION PROCESS** – The organization of services for an optimal management of the patient in all phases of the disorder, with focus on the transition process in the adolescent schizophrenic patient: optimal procedures and areas of improvement in the collaboration between the services of child/adolescent and adult psychiatry	17 – A service dedicated to identifying and treating subjects at risk or those affected by an initial psychotic episode must:		
	17.1 – present characteristics of personalisation and specificity of the interventions	0%	100%
	17.2 – be able to offer suitable informative activities to the general practitioners to the regional health service operators and to the population	1%	99%
	18 – Taking in charge of “complex cases”, in particular those medical comorbidities, must also include in the treatment team:		
	18.1 – Neuropsychiatry	4%	96%
	18.2 – Adult psychiatrist	9%	91%
	18.3 – Psychiatrist expert in substance abuse	13%	87%
	18.4 – Case manager recognized as the contact person in the course of cure and trade union during the transition process	0%	100%
	19 – In order to favor access to the mental health services of adolescent and young adult patients with schizophrenia, it would be necessary to:		
	19.1 – Promote the realization of specific protocols of collaboration in the field (between services of Child/Adolescent Psychiatry, mental health for adults and of Substance Abuse)	1%	99%
	19.2 – Promote the realization of specific protocols of collaboration between the services of mental health for adolescents, pediatricians and general practitioners	3%	97%
	19.3 – Promote a close relationship between the mental health services for adolescents and areas of youth aggregation (school, social services, associations, sports)	1%	99%
	19.4 – Ensure the continuity of the cure in the transition between the services of the Child/Adolescent psychiatry and the services of mental health for adults by means of multidisciplinary specialist teams	0%	100%
	19.5 – Favor the knowledge of mental health problems by means of “new” means of communication, such as Internet and social networks.	1%	99%
	19.6 – Guarantee the presence of services of the child/adolescent psychiatry in every region	0%	100%
	19.7 – Make material on SCZ available to the patient and their family/caregivers	4%	96%
**PSYCHOSOCIAL INTERVENTIONS** – Role of psychosocial interventions in support to pharmacological therapy in youth with schizophrenia	20 - For adolescents with schizophrenia, psychoeducation should always be directed to both the patients and their caregivers, and to their peers	0%	100%
	21 - Psychotherapeutic interventions, social skills training, cognitive rehabilitation and family interventions should be provided to all patients, but should not be considered alternatives to pharmacotherapy	0%	100%

*DUP, Duration of Untreated Psychosis; SCZ, schizophrenia*.

**Table 2.2:** Mean, median, mode and standard deviation of the consensus statement.

	**Strongly disagree**	**Disagree**	**Agree**	**More than agree**	**Strongly agree**		**Weighted mean**	**Standard deviation**	**Median**	**Mode**	**Weighted mean**	**Standard deviation**	**Median**	**Mode**
	**1**	**2**	**3**	**4**	**5**									
Statement 1	0	1	1	10	58	70	0.7	0.7	0.5	#N/D	27.8	30.6	10.0	#N/D
Tot disagree/agree	1.4%		98.6%			100.0%								
Statement 2	0	1	5	17	47	70	0.7	0.7	0.5	#N/D	26.5	21.6	17.0	#N/D
Tot disagree/agree	1.4%		98.6%			100.0%								
Statement 3	0	1	8	30	31	70	0.7	0.7	0.5	#N/D	24.9	13.0	30.0	#N/D
Tot disagree/agree	1.4%		98.6%			100.0%								
Statement 4.1	1	4	7	33	25	70	3.0	2.1	2.5	#N/D	23.2	13.3	25.0	#N/D
Tot disagree/agree	7.1%		92.9%			100.0%								
Statement 4.2	0	3	13	28	26	70	2.0	2.1	1.5	#N/D	23.4	8.1	26.0	#N/D
Tot disagree/agree	4.3%		95.7%			100.0%								
Statement 4.3	0	11	10	30	19	70	7.3	7.8	5.5	#N/D	20.4	10.0	19.0	#N/D
Tot disagree/agree	15.7%		84.3%			100.0%								
Statement 4.4	0	9	15	26	20	70	6.0	6.4	4.5	#N/D	20.8	5.5	20.0	#N/D
Tot disagree/agree	12.9%		87.1%			100.0%								
Statement 4.5	2	9	20	22	17	70	6.7	4.9	5.5	#N/D	19.4	2.5	20.0	#N/D
Tot disagree/agree	15.7%		84.3%			100.0%								
Statement 5	0	2	5	27	36	70	1.3	1.4	1.0	#N/D	25.3	15.9	27.0	#N/D
Tot disagree/agree	2.9%		97.1%			100.0%								
Statement 6.1	0	1	9	17	43	70	0.7	0.7	0.5	#N/D	25.8	17.8	17.0	#N/D
Tot disagree/agree	1.4%		98.6%			100.0%								
Statement 6.2	0	0	13	22	35	70	0.0	0.0	0.0	0.0	25.2	11.1	22.0	#N/D
Tot disagree/agree	0.0%		100.0%			100.0%								
Statement 6.3	2	7	25	17	19	70	5.3	3.5	4.5	#N/D	19.8	4.2	19.0	#N/D
Tot disagree/agree	12.9%		87.1%			100.0%								
Statement 6.4	1	4	25	17	23	70	3.0	2.1	2.5	#N/D	21.5	4.2	23.0	#N/D
Tot disagree/agree	7.1%		92.9%			100.0%								
Statement 6.5	0	10	25	20	15	70	6.7	7.1	5.0	#N/D	19.2	5.0	20.0	#N/D
Tot disagree/agree	14.3%		85.7%			100.0%								
Statement 6.6	0	5	15	25	25	70	3.3	3.5	2.5	#N/D	22.5	5.8	25.0	25.0
Tot disagree/agree	7.1%		92.9%			100.0%								
Statement 6.7	0	4	15	25	26	70	2.7	2.8	2.0	#N/D	22.9	6.1	25.0	#N/D
Tot disagree/agree	5.7%		94.3%			100.0%								
Statement 6.8	0	0	7	22	41	70	0.0	0.0	0.0	0.0	26.2	17.0	22.0	#N/D
Tot disagree/agree	0.0%		100.0%			100.0%								
Statement 7	5	24	23	6	12	70	17.7	13.4	14.5	#N/D	12.8	8.6	12.0	#N/D
Tot disagree/agree	41.4%		58.6%			100.0%								
Second round-revision Statement 7a	10	39	13	3	5	70	29.3	20.5	24.5	#N/D	6.3	5.3	5.0	#N/D
Tot disagree/agree	70.0%		30.0%			100.0%								
Second round-revision Statement 7b	0	3	8	19	40	70	2.0	2.1	1.5	#N/D	25.0	16.3	19.0	#N/D
Tot disagree/agree	4.3%		95.7%			100.0%								
Statement 8	1	3	20	17	29	70	2.3	1.4	2.0	#N/D	22.8	6.2	20.0	#N/D
Tot disagree/agree	5.7%		94.3%			100.0%								
Statement 9.1	0	0	5	14	51	70	0.0	0.0	0.0	0.0	27.2	24.4	14.0	#N/D
Tot disagree/agree	0.0%		100.0%			100.0%								
Statement 9.2	1	2	11	19	37	70	1.7	0.7	1.5	#N/D	24.5	13.3	19.0	#N/D
Tot disagree/agree	4.3%		95.7%			100.0%								
Statement 9.3	0	0	11	19	40	70	0.0	0.0	0.0	0.0	25.8	15.0	19.0	#N/D
Tot disagree/agree	0.0%		100.0%			100.0%								
Statement 9.4	0	1	6	15	48	70	0.7	0.7	0.5	#N/D	26.5	22.1	15.0	#N/D
Tot disagree/agree	1.4%		98.6%			100.0%								
Statement 9.5	0	0	7	17	46	70	0.0	0.0	0.0	0.0	26.6	20.3	17.0	#N/D
Tot disagree/agree	0.0%		100.0%			100.0%								
Statement 10	0	3	7	22	38	70	2.0	2.1	1.5	#N/D	24.9	15.5	22.0	#N/D
Tot disagree/agree	4.3%		95.7%			100.0%								
Statement 11	0	0	10	18	42	70	0.0	0.0	0.0	0.0	26.0	16.7	18.0	#N/D
Tot disagree/agree	0.0%		100.0%			100.0%								
Statement 8	1	3	20	17	29	70	2.3	1.4	2.0	#N/D	22.8	6.2	20.0	#N/D
Tot disagree/agree	5.7%		94.3%			100.0%								
Statement 9.1	0	0	5	14	51	70	0.0	0.0	0.0	0.0	27.2	24.4	14.0	#N/D
Tot disagree/agree	0.0%		100.0%			100.0%								
Statement 9.2	1	2	11	19	37	70	1.7	0.7	1.5	#N/D	24.5	13.3	19.0	#N/D
Tot disagree/agree	4.3%		95.7%			100.0%								
Statement 9.3	0	0	11	19	40	70	0.0	0.0	0.0	0.0	25.8	15.0	19.0	#N/D
Tot disagree/agree	0.0%		100.0%			100.0%								
Statement 9.4	0	1	6	15	48	70	0.7	0.7	0.5	#N/D	26.5	22.1	15.0	#N/D
Tot disagree/agree	1.4%		98.6%			100.0%								
Statement 9.5	0	0	7	17	46	70	0.0	0.0	0.0	0.0	26.6	20.3	17.0	#N/D
Tot disagree/agree	0.0%		100.0%			100.0%								
Statement 10	0	3	7	22	38	70	2.0	2.1	1.5	#N/D	24.9	15.5	22.0	#N/D
Tot disagree/agree	4.3%		95.7%			100.0%								
Statement 11	0	0	10	18	42	70	0.0	0.0	0.0	0.0	26.0	16.7	18.0	#N/D
Tot disagree/agree	0.0%		100.0%			100.0%								
Statement 12.1	0	3	11	19	37	70	2.0	2.1	1.5	#N/D	24.5	13.3	19.0	#N/D
Tot disagree/agree	4.3%		95.7%			100.0%								
Statement 12.2	0	4	21	24	21	70	2.7	2.8	2.0	#N/D	22.0	1.7	21.0	21.0
Tot disagree/agree	5.7%		94.3%			100.0%								
Statement 12.3	0	0	5	13	52	70	0.0	0.0	0.0	0.0	27.3	25.1	13.0	#N/D
Tot disagree/agree	0.0%		100.0%			100.0%								
Statement 12.4	0	0	1	18	51	70	0.0	0.0	0.0	0.0	27.5	25.4	18.0	#N/D
Tot disagree/agree	0.0%		100.0%			100.0%								
Statement 12.5	0	4	19	23	24	70	2.7	2.8	2.0	#N/D	22.4	2.6	23.0	#N/D
Tot disagree/agree	5.7%		94.3%			100.0%								
Statement 12.6	0	0	7	20	43	70	0.0	0.0	0.0	0.0	26.3	18.2	20.0	#N/D
Tot disagree/agree	0.0%		100.0%			100.0%								
Statement 12.7	0	1	2	16	51	70	0.7	0.7	0.5	#N/D	27.1	25.2	16.0	#N/D
Tot disagree/agree	1.4%		98.6%			100.0%								
Statement 13.1	0	0	14	16	40	70	0.0	0.0	0.0	0.0	25.5	14.5	16.0	#N/D
Tot disagree/agree	0.0%		100.0%			100.0%								
Statement 13.2	0	10	25	23	12	70	6.7	7.1	5.0	#N/D	18.9	7.0	23.0	#N/D
Tot disagree/agree	14.3%		85.7%			100.0%								
Statement 13.3	0	3	23	16	28	70	2.0	2.1	1.5	#N/D	22.8	6.0	23.0	#N/D
Tot disagree/agree	4.3%		95.7%			100.0%								
Statement 13.4	0	0	7	32	31	70	0.0	0.0	0.0	0.0	25.3	14.2	31.0	#N/D
Tot disagree/agree	0.0%		100.0%			100.0%								
Statement 13.5	1	8	21	21	19	70	5.7	4.9	4.5	#N/D	20.2	1.2	21.0	21.0
Tot disagree/agree	12.9%		87.1%			100.0%								
Statement 14	0	20	24	18	8	70	13.3	14.1	10.0	#N/D	15.3	8.1	18.0	#N/D
Tot disagree/agree	28.6%		71.4%			100.0%								
Statement 15.1	0	1	1	19	49	70	0.7	0.7	0.5	#N/D	27.0	24.2	19.0	#N/D
Tot disagree/agree	1.4%		98.6%			100.0%								
Statement 15.2	0	10	19	24	17	70	6.7	7.1	5.0	#N/D	19.8	3.6	19.0	#N/D
Tot disagree/agree	14.3%		85.7%			100.0%								
Statement 15.3	0	0	3	20	47	70	0.0	0.0	0.0	0.0	27.0	22.2	20.0	#N/D
Tot disagree/agree	0.0%		100.0%			100.0%								
Statement 15.4	0	2	20	25	23	70	1.3	1.4	1.0	#N/D	22.9	2.5	23.0	#N/D
Tot disagree/agree	2.9%		97.1%			100.0%								
Statement 15.5	6	23	20	10	11	70	17.3	12.0	14.5	#N/D	12.9	5.5	11.0	#N/D
Tot disagree/agree	41.4%		58.6%			100.0%								
Second round - revision (15.5) Statement 15.5.1	6	47	11	6	0	70	33.3	29.0	26.5	#N/D	4.8	5.5	6.0	#N/D
Tot disagree/agree	75.7%		24.3%			100.0%								
Statement 15.5.2	6	39	15	8	2	70	28.0	23.3	22.5	#N/D	7.3	6.5	8.0	#N/D
Tot disagree/agree	64.3%	35.7%	100.0%								
Statement 15.5.3	2	18	28	14	8	70	12.7	11.3	10.0	#N/D	15.0	10.3	14.0	#N/D
Tot disagree/agree	28.6%	71.4%	100.0%								
Statement 15.5.4	3	12	30	9	16	70	9.0	6.4	7.5	#N/D	17.2	10.7	16.0	#N/D
Tot disagree/agree	21.4%	78.6%	100.0%								
Statement 15.6	0	0	8	25	37	70	0.0	0.0	0.0	0.0	25.8	14.6	25.0	#N/D
Tot disagree/agree	0.0%	100.0%	100.0%								
Statement 15.7	0	1	1	13	55	70	0.7	0.7	0.5	#N/D	27.5	28.4	13.0	#N/D
Tot disagree/agree	1.4%	98.6%	100.0%								
Statement 15.8	1	4	20	17	28	70	3.0	2.1	2.5	#N/D	22.3	5.7	20.0	#N/D
Tot disagree/agree	7.1%	92.9%	100.0%								
Statement 16.1	0	7	11	25	27	70	4.7	4.9	3.5	#N/D	22.3	8.7	25.0	#N/D
Tot disagree/agree	10.0%	90.0%	100.0%								
Statement 16.2	0	1	11	25	33	70	0.7	0.7	0.5	#N/D	24.8	11.1	25.0	#N/D
Tot disagree/agree	1.4%	98.6%	100.0%								
Statement 16.3	0	2	16	22	30	70	1.3	1.4	1.0	#N/D	23.8	7.0	22.0	#N/D
Tot disagree/agree	2.9%	97.1%	100.0%								
Statement 16.4	0	1	14	21	34	70	0.7	0.7	0.5	#N/D	24.7	10.1	21.0	#N/D
Tot disagree/agree	1.4%	98.6%	100.0%								
Statement 17.1	0	0	0	20	50	70	0.0	0.0	0.0	0.0	27.5	25.2	20.0	#N/D
Tot disagree/agree	0.0%	100.0%	100.0%								
Statement 17.2	0	1	4	21	44	70	0.7	0.7	0.5	#N/D	26.3	20.1	21.0	#N/D
Tot disagree/agree	1.4%	98.6%	100.0%								
Statement 18.1	0	3	5	13	49	70	2.0	2.1	1.5	#N/D	26.0	23.4	13.0	#N/D
Tot disagree/agree	4.3%		95.7%			100.0%								
Statement 18.2	0	6	15	18	31	70	4.0	4.2	3.0	#N/D	22.7	8.5	18.0	#N/D
Tot disagree/agree	8.6%		91.4%			100.0%								
Statement 18.3	0	9	19	22	20	70	6.0	6.4	4.5	#N/D	20.4	1.5	20.0	#N/D
Tot disagree/agree	12.9%		87.1%			100.0%								
Statement 18.4	0	0	6	13	51	70	0.0	0.0	0.0	0.0	27.1	24.2	13.0	#N/D
Tot disagree/agree	0.0%		100.0%			100.0%								
Statement 19.1	0	1	1	23	45	70	0.7	0.7	0.5	#N/D	26.7	22.0	23.0	#N/D
Tot disagree/agree	1.4%		98.6%			100.0%								
Statement 19.2	0	2	4	22	42	70	1.3	1.4	1.0	#N/D	25.8	19.0	22.0	#N/D
Tot disagree/agree	2.9%		97.1%			100.0%								
Statement 19.3	0	1	9	25	35	70	0.7	0.7	0.5	#N/D	25.2	13.1	25.0	#N/D
Tot disagree/agree	1.4%		98.6%			100.0%								
Statement 19.4	0	0	2	15	53	70	0.0	0.0	0.0	0.0	27.6	26.5	15.0	#N/D
Tot disagree/agree	0.0%		100.0%			100.0%								
Statement 19.5	0	1	19	30	20	70	0.7	0.7	0.5	#N/D	23.1	6.1	20.0	#N/D
Tot disagree/agree	1.4%		98.6%			100.0%								
Statement 19.6	0	0	3	15	52	70	0.0	0.0	0.0	0.0	27.4	25.5	15.0	#N/D
Tot disagree/agree	0.0%		100.0%			100.0%								
Statement 19.7	0	3	11	26	30	70	2.0	2.1	1.5	#N/D	23.9	10.0	26.0	#N/D
Tot disagree/agree	4.3%		95.7%			100.0%								
Statement 20	0	0	5	24	41	70	0.0	0.0	0.0	0.0	26.3	18.0	24.0	#N/D
Tot disagree/agree	0.0%		100.0%			100.0%								
Statement 21	0	0	8	18	44	70	0.0	0.0	0.0	0.0	26.3	18.6	18.0	#N/D
Tot disagree/agree	0.0%		100.0%			100.0%								

*#N/D, dataset does not contain duplicate values*.

Major statements, grouped in the four macro-areas, are reported below.

### Early Diagnosis

This area includes eight statements and 19 items, concerning: the DUP, the importance of an early diagnosis and an early intervention, the diagnostic process, the use of assessment tools, and the challenges and the barriers for an early recognition. In the second round of the survey, one statement of this investigating area (statement - 7) was split in two items. More in detail, a high consensus was reached in the following items: reducing DUP, recognizing adolescents at risk of developing psychosis and in prodromal phase, investigating the presence of other neurodevelopment disorders and other comorbidity using standardized and validated assessment tools, and hospitalization (if necessary) only in structures specifically dedicated for this development stage. Furthermore, a broad consensus was obtained regarding the barriers and obstacles in recognition and in early identification of subjects with adolescent schizophrenia, such as the lack of human resources and trained operators, the scarcity of investments and specific dedicated programs and the adverse role of stigma. On the other hand, a negative consensus was expressed regarding the need of hospitalization in any patients with first-episode psychosis.

### Pharmacological Treatment

This area includes eight statements and 32 items, concerning: the choice of antipsychotic treatment, FGAs and SGAs, the use of off-label drugs, the efficacy on positive, negative and cognitive symptoms, side effects, safety, the duration of treatment, the presence of comorbidities, a concomitant substance abuse, when considering a switch, clinical and functional outcome, patient wellbeing, and family involvement. In the second round of the survey, one statement of this investigating area (statement – 15; item 15.5) was edited into four items. In particular, a broad consensus was achieved in the following items: the importance of the choice of antipsychotic treatments on the basis of the clinical characteristics, such as the stage of the illness, the presence of medical comorbidities and eventual concomitant substance abuse, also taking into account side effects, safety, and the characteristics of the drug, such as the preference for SGAs, and the efficacy on positive, negative, and cognitive symptoms. Furthermore, a large consensus was also obtained regarding the need of a continuous pharmacological treatment, shared with the patient and the family, better if integrated with psychosocial interventions, considering not only the symptomatic, but also the functional remission and the subjective wellbeing. A broad agreement was additionally expressed concerning the off-label drugs utilization, the utility of clozapine in resistant patients, and when considering a switch to other antipsychotics. Concerning this last point, a high consensus was achieved regarding the need of a switch in cases of reduced clinical efficacy and poor tolerability, including metabolic side effects, the presence of medical comorbidities and a high suicidal risk. Instead, the experts displayed a lack of or a negative consensus about the opportunity to stop an antipsychotic therapy. More specifically, they seem to advise against a suspension of antipsychotics after 1 year of treatment, even if there is a clinical and/or functional remission, while they show some agreement on the possibility to stop antipsychotics after at least 2 years of treatment, only if there is a clinical and/or functional remission.

### Health Care System Organization and Transition Process From Adolescent to Adulthood

This area includes three statements and 13 items, concerning: the characteristics of a mental health service for adolescents, the multidisciplinary team-working, the role of the case manager, how to facilitate access to mental health services, how to promote transition between child neuropsychiatry and adult psychiatry, and how to ensure continuity of care in the early phases of schizophrenia. The experts considered the need to implement a service dedicated to the early identification and early intervention, which would operate in close collaboration with the local services, including general practitioners and population. Furthermore, the management of complex patients should include a joint and integrated work between the services of child neuropsychiatry and adult psychiatry, also including the services for addiction and identifying a case manager. Finally, a broad consensus was also reached with respect to the characteristics required to facilitate the access to the mental health services for adolescents and young adults. More specifically, it would be necessary to implement some collaboration protocols between child neuropsychiatry services, adult mental health services, addiction services, pediatricians, and general practitioners, in order to ensure a continuity of care in the early phases of schizophrenia and to promote the process of transition between child neuropsychiatry and adult psychiatry. This process could be facilitated implementing specialized multidisciplinary cross teams to the different services. Full consensus was also demonstrated regards the need to ensure the presence of child neuropsychiatry services in every territory at a national level. Lastly, it emerged how important it is to give a particular attention to the correct dissemination of information to patients and family members, also through the new social media, as well as promoting a close relationship with the areas of youth aggregation.

### Psychosocial Interventions

This area includes two statements, concerning: psychoeducational interventions for the patient and for the family, evidence-based rehabilitation interventions, and integrated treatments. This area of investigation also obtained a full consensus. In particular among rehabilitation interventions, those evidence-based, such as psychoeducation, social skills training, and cognitive rehabilitation, should be taken into account.

## Discussion

A two-step Delphi approach was used to share evidence-based information on early-onset/adolescent schizophrenia management, assessing the degree of consensus among specialists in psychiatry and child neuropsychiatry on the following key issues: (i) early diagnosis; (ii) pharmacological treatment; (iii) health care system organization as well as transition process from adolescent to adulthood; and (iv) psychosocial interventions. The results of the survey revealed a large consensus among the experts on all the investigated areas of adolescent schizophrenia patterns of care and management.

A high consensus was reached in the first area of investigation about early diagnosis and intervention and, particularly, the importance of the following variables: reducing DUP, recognizing adolescents at risk of developing psychosis and in prodromal phase, investigating the presence of other neurodevelopment disorders and other comorbidity using standardized and validated assessment tools, and hospitalization (if necessary) only in structures specifically dedicated for this development stage. Furthermore, a broad consensus was obtained regarding the barriers and obstacles in recognition and in early identification of subjects with adolescent schizophrenia, such as the lack of human resources and trained operators, the scarcity of investments and specific dedicated programs and the adverse role of stigma. These results show how psychiatrists and child neuropsychiatrists, in accordance with the scientific literature, and ministerial and regional recommendations, are completely aware of the importance of early recognition and intervention, even in subjects at risk of psychosis and in the prodromal phases (16, 17, 60). However, this approach is not always applied and is not feasible throughout the national territory, due to some barriers identified by the survey.

On the other hand, a negative consensus was expressed regarding the need of hospitalization in any patients with first-episode psychosis. This interesting result, in agreement with national and international literature (64, 65), could mean the necessity and the awareness of the experts to implement a community intervention, not based only on hospitalization.

The survey demonstrated a broad consensus in the second area of investigation that is the pharmacological treatment. In particular, it was outlined the importance of the choice of antipsychotic treatments on the basis of the clinical characteristics of the patient, such as the stage of the illness, the presence of medical comorbidities and eventual concomitant substance abuse. Side effects and the characteristics of the drug, such as the preference for SGAs, the efficacy on positive, negative and cognitive symptoms and the safety must be also considered. Furthermore, a large consensus was also obtained regarding the need of a continuous pharmacological treatment, shared with the patient and the family, better if integrated with psychosocial interventions, considering not only the symptomatic, but also the functional remission and the subjective wellbeing. A broad agreement was additionally expressed concerning the off-label drugs utilization (an option which could be considered, for example, for adolescent patients with demonstrated non-adherence to approved antipsychotics or in those with reduced clinical response or occurrence of adverse effects associated with approved antipsychotics), the utility of clozapine in resistant patients, and when considering a switch to other antipsychotics. In particular, for this last point, a high consensus was achieved regarding the need of a switch in cases of reduced clinical efficacy and poor tolerability, including metabolic side effects, the presence of medical comorbidities and a high suicidal risk. Regarding this area of investigation, results show that the choice of an antipsychotic therapy requires a careful and comprehensive patient assessment, giving attention not only to the symptomatic remission, but also to the functional remission and subjective wellbeing (35). In this regard, it is relevant to remind that the aim of schizophrenia treatment is not the remission of specific psychotic symptoms, but the improvement of functional outcomes and quality of life, always keeping in mind patient goals (66). Moreover, the risk/benefit ratio between effectiveness and side effects should be weighted for each patient (i.e., the long-term risk of metabolic side effects) (67). It should be emphasized how these results are in line with the international literature (15, 68–70). Instead, the experts displayed a lack of or a negative consensus about the opportunity to stop an antipsychotic therapy. More specifically, they seem to advise against a suspension of antipsychotics after 1 year of treatment, even if there is a clinical and/or functional remission, while they show some agreement on the possibility to stop antipsychotics after at least 2 years of treatment, only if there is a clinical and/or functional remission. It is interesting to note that this result is in any case consistent with the non-definitive and controversial international scientific literature on this still debated topic (71–74). The duration of maintenance treatment following a first-episode of schizophrenia is one of the most debated issue in the treatment of youth with schizophrenia, that has been addressed in a well-conducted systematic review performed by Keating et al. (72). In this regard, although good quality guidelines to assist in pharmacological treatment optimization exist, authors pointed out the inconsistencies between guidelines after the first-episode, underlining that the evidence base required to answer key health questions relevant to the pharmacological treatment of first-episode schizophrenia is still limited.

The third investigated area on the health care system organization and transition process from adolescent to adulthood reached high rates of consensus. In particular, the experts considered the need to implement a service, with specific characteristics, dedicated to the early identification and early intervention, which would operate in close collaboration with the local services, including general practitioners and population. Furthermore, the management of complex patients should include a joint and integrated work between the services of child neuropsychiatry and adult psychiatry, also including the services for addiction and identifying a case manager. Finally, a broad consensus was also reached with respect to the characteristics required to facilitate the access to the mental health services for adolescents and young adults. More specifically, it would be necessary to implement some collaboration protocols between child neuropsychiatry services, adult mental health services, addiction services, pediatricians, and general practitioners, in order to ensure a continuity of care in the early phases of schizophrenia and to promote the process of transition between child neuropsychiatry and adult psychiatry. This process could be facilitated implementing specialized multidisciplinary cross teams to the different services. Full consensus was also demonstrated regards the need to ensure the presence of child neuropsychiatry services in every territory at a national level. Lastly, it emerged how important it is to give a particular attention to the correct dissemination of information to patients and family members, also through the new social media, as well as promoting a close relationship with the areas of youth aggregation. Overall, in line with the existing scientific literature (75), this area of investigation about the organization of mental health services and on the transition process from adolescent to adulthood obtained a broad consensus among experts. Despite this agreement, the mental health services reality is not always the same expressed in the survey results and the dedicated services for early recognition and intervention, especially in the acute phases, are not uniformly spread throughout the national territory. Furthermore, these services, when existing, are not always well-integrated with each other, and do not work in multidisciplinary teams, therefore without promoting a stable patient health care path and a suitable transition between neuropsychiatry and adult psychiatry.

The fourth area of investigation obtained a full consensus on the use of integrated psychotherapeutic and psychosocial interventions. Among rehabilitation interventions, those evidence-based should be taken into account, in particular psychoeducation aimed at both patients and family members, social skills training, and cognitive rehabilitation. Again, the experts' panel was absolutely in agreement with the scientific literature (76–78). As in previous research fields taken into account in the present survey, such as the implementation of early recognition and identification services, evidence-based rehabilitation interventions and integrated treatments showed a significant gap between the acquired scientific knowledge and clinical practice. This means that, despite the growing scientific literature on these topics, a major concern is that evidence-based rehabilitation interventions and integrated treatments are not largely available in the real-world setting of mental health services (79).

Lastly, cost-effectiveness is an issue common to all the four investigated areas. In fact, both for early diagnosis, pharmacological and psychosocial treatments, as well as health care system organization, it is relevant to consider resources. In the real-world setting of adolescents and adults Mental Health Services, adequate resources for implementing the best clinical-therapeutic practices are not always available. In this scenario, even if psychotherapeutic and psychoeducational approaches may be helpful, but with very low evidence in youth (80), pharmacotherapy should always be the first option, even when other interventions are unavailable. However, it should also be highlighted that many psychosocial interventions, such as psychoeducation (81) and other integrated interventions (82), may be both clinically beneficial and cost-effectiveness in the early stages of the schizophrenia spectrum disorders.

### Limitations

In this project some limitations are to be addressed. First, adherence to drug therapy and in particular the use of long-acting injectable (LAI) antipsychotics was not directly investigated. Second, the results may not be representative of the entire national territory and may not be generalizable to other Countries. Third, the survey involved only specialists in child neuropsychiatry and in adult psychiatry, but not pediatricians, general practitioners, other mental health workers, and patients and family association members, so it does not cover the views of all the stakeholders involved in this complex field.

## Conclusion

To our knowledge, this is the first Delphi-based consensus survey on patterns of care in adolescent schizophrenia, involving experts and specialists in child neuropsychiatry and in adult psychiatry. The results of this consensus Delphi approach revealed a large agreement among the expert group of Italian specialists in child neuropsychiatry and in adult psychiatry on all the investigated areas of adolescent schizophrenia patterns of care and management. In particular, the level of agreement was maximum in the following crucial issues: early diagnosis; pharmacological treatment; health care system organization as well as transition process from adolescent to adulthood; and psychosocial interventions. Overall, results showed a significant gap between the acquired scientific knowledge and clinical practice. This means that, despite the growing scientific literature on early-onset/adolescent schizophrenia management, a major concern is that knowledge is not largely available in the real-world setting of mental health services. Of particular interest is that this science-to-service gap seems to be well-recognized by Italian specialists, who identify the lack of human resources and trained operators and the paucity of investments and specific dedicated programs as the main barriers that do not allow to fill the gap.

In this scenario, it should be necessary to program specific training events on adolescent/early onset schizophrenia and on the transition process, to draft and spread agreement protocols between child neuropsychiatry and adult psychiatry and to set up multidisciplinary teams transversal to services. Moreover, appear crucial to edit recommendations on clinical decision-making as well as to prompt changes at the political and organizational levels, also involving scientific societies, patients and family associations and all the stakeholders involved, in order to overcome the barriers that delay the implementation process.

## Data Availability Statement

The original contributions presented in the study are included in the article/supplementary material, further inquiries can be directed to the corresponding author/s.

## Ethics Statement

The study was based on a survey that does not involve the participation of human subjects nor patient data management. Consequently, this study did not require ethical approval. All experts involved in the Delphi survey were informed of the study's objectives and the possibility of publishing the results in a peer-reviewed article. The participation was voluntary.

## Author Contributions

All authors contributed to the writing and editing of the manuscript. All authors approved the final version of the manuscript.

## Funding

GM was supported by funds of the Italian Ministry of Health: Ricerca Corrente (Project 2.10, PI Gabriele Masi). The authors declare that this study received funding from Angelina Pharma. The funder was not involved in the study design, collection, analysis, interpretation of data, the writing of this article or the decision to submit it for publication.

## Conflict of Interest

The authors declare that the research was conducted in the absence of any commercial or financial relationships that could be construed as a potential conflict of interest.

## Publisher's Note

All claims expressed in this article are solely those of the authors and do not necessarily represent those of their affiliated organizations, or those of the publisher, the editors and the reviewers. Any product that may be evaluated in this article, or claim that may be made by its manufacturer, is not guaranteed or endorsed by the publisher.
